# A Systematic Review of the Efficacy and Safety of Tenecteplase Versus Streptokinase in the Management of Myocardial Infarction in Developing Countries

**DOI:** 10.7759/cureus.44125

**Published:** 2023-08-25

**Authors:** Chioma G Muoghalu, Ndianabasi Ekong, William Wyns, Cosmas C Ofoegbu, Micheal Newell, Danvictor A Ebirim, Sandra T Alex-Ojei

**Affiliations:** 1 Department of Medicine, University of Galway, Galway, IRL; 2 Department of Medicine, Medical Center, Akwa Ibom State College of Education, Afaha Nsit, NGA; 3 Department of Public Health, General Hospital, Jedda, SAU; 4 Department of Surgery, University of Galway, Galway, IRL; 5 Department of Medicine, Federal Medical Center, Owerri, NGA; 6 Department of Medicine, University of Port Harcourt Teaching Hospital, Port Harcourt, NGA

**Keywords:** tenecteplase, tnk, tnkase, streptokinase, sk, stk, ami, myocardial infarction, heart infarction, stemi

## Abstract

Myocardial infarction (MI) is a significant cause of morbidity and mortality in low- and middle-income countries. Fibrinolytic agents and percutaneous coronary intervention (PCI) are the main approaches for the recanalization and reperfusion of the myocardium following MI. Many studies have shown that PCI is superior to thrombolytics due to better outcomes and decreased mortality. Nevertheless, PCI's mortality gain over thrombolysis decreases as the time between presentation and PCI procedure increases. Furthermore, PCI is not widely available in most developing countries; thus, it cannot be delivered promptly. Most patients in developing countries cannot afford the cost of PCI. Thus, thrombolytic therapy remains essential to managing MI in developing countries and should not be disregarded. Tenecteplase (TNK) and streptokinase (SK) are the two most widely used fibrinolytics in managing MI in underdeveloped nations. Despite their widespread availability, comparative studies on them have been inconclusive. This study aims to review the available literature on the effectiveness and safety of TNK versus SK in managing MI in resource-poor nations.

The study is reported according to the Preferred Reporting Items for Systematic Reviews and Meta-analysis (PRISMA) extension and analyzed according to Cochrane guidelines on synthesis without meta-analysis. A comprehensive literature search for studies comparing TNK and STK was conducted on EMBASE, Cochrane Library, Web of Science, CINAHL, Scopus, Google Scholar, and Ovid version of MEDLINE databases. A reference list of the eligible articles and systematic reviews was also screened. A narrative synthesis of the available data was done by representing the data on the effect direction plot, followed by vote counting.

Of the 2284 references retrieved from the databases, only 17 studies met the inclusion criteria and were selected for final analysis. The study suggested that TNK is more effective in complete ST-segment resolution (80% vs 10% on the effect direction plot) and symptom relief (80% vs 20%) than SK. SK and TNK were comparable in achieving successful fibrinolysis (50% vs 50%). For the safety parameters, TNK is associated with a lesser risk of major bleeding than SK (88.9% vs 11.1%) and minor bleeding (25% vs 75%). SK was linked with a higher risk of hypotension/shock (77.8% vs 11.1%) and anaphylaxis/allergy (100% vs 0%). Long-term mortality was higher in the SK arm (100% vs 0%). In-hospital mortality is comparable between the two agents (37.5% vs 37.5%). There is conflicting evidence regarding other safety and efficacy endpoints.

Compared to SK, TNK results in better complete ST-segment resolution and symptom relief. A higher risk of long-term mortality, increased risk of major and minor bleeding, hypotension, and allergy/anaphylaxis was observed in patients who received SK. Both agents were comparable in terms of in-hospital mortality and successful fibrinolysis. Controversy exists regarding which agent is linked with increased risk of 30-35-day mortality benefit and stroke. Randomized controlled trials (RCTs) with large sample sizes are needed to establish TNK vs SK superiority in efficacy and safety. The long-term duration of follow-up of the mortality rate of the two agents is also essential, as most patients in these regions cannot afford the recommended PCI post-fibrinolysis.

## Introduction and background

Worldwide, the leading cause of death is cardiovascular disease (CVD) [1]. In 2019, about 17.9 million people died from CVDs, and 85% of these deaths were due to myocardial infarction (MI) [1]. Over 75% of CVD deaths occur in developing countries. Similarly, MI is the third leading cause of death in developing countries [1]. Acute MI is a life-threatening condition initiated by pathological cascades of events starting with plaque rupture in the coronary arteries leading to thrombus formation, occlusion of the vessel, and culminating in ischemia and eventual infarction of the myocardium if recanalization is not achieved on time [2]. Hence, early achievement of coronary vessel patency is critically vital in improving mortality, limiting infarct size and subsequent left ventricular dysfunction.

Currently, the main approaches for achieving recanalization and reperfusion of the myocardium are fibrinolytic agents and percutaneous coronary intervention (PCI) [3]. Many studies have shown that PCI is superior to thrombolytics due to better outcomes and decreased mortality [3]. Comparably, a meta-analysis of 10 randomized controlled trials (RCTs) reported that PCI is associated with improved 30-day mortality compared with fibrinolytic agents [4]. Nevertheless, PCI's mortality gain over thrombolysis decreases as the time between presentation and PCI procedure increases [5]. A PCI approach may not reduce mortality when a delay of more than 60 minutes is expected versus instant utilization of fibrinolytic therapy [5]. Based on the available studies, it is not plausible to conclude that a particular treatment approach is better for every patient at all times and in every clinical setup [3]. Thus, the American Heart Association (AHA) suggests that the appropriate and immediate use of available reperfusion therapy is more important than treatment choice [5]. Indeed, the European cardiology guideline recommends PCI only when it can be achieved within two hours [3]. Likewise, AHA recommends PCI only when specific criteria are met (door-to-balloon time <90 minutes, skill PCI laboratory with available surgical backup, etc.) [5].

PCI is not readily available in developing countries in all healthcare settings [6]. In India, less than 10% of patients presenting with MI are treated with PCI due to the unavailability of cath labs and transportation delays [7]. The same applies to most developing countries. On the other hand, those who present early have insufficient funds because of a lack of health insurance [7]. Patients in most resource-poor nations pay out of pocket for hospital bills, and most hospitals that offer PCI procedures belong to the private sector without influence from the central government [7]. The Acute Coronary Event Strategies Survey (ACCESS Registry) trial conducted in nine developing countries noted that only 20% of cases of ST-elevation MI (STEMI) are managed with PCI [6]. Likewise, the GULF Race study in Asia reported that 93% of MI cases were managed with fibrinolytic, whereas 7% had PCI [8].

In contrast to PCI, doctors can administer fibrinolytics with little or no experience, and it's not operator-dependent. RCTs have reported that initiating fibrinolytics early after symptom onset leads to a high reperfusion rate and improves mortality [5]. A more significant proportion of lives could be saved if fibrinolytics could be commenced at the time of prehospital or first-hospital evaluation, especially if initiated within one hour of symptom onset [5]. Generally, fibrinolytics can be provided earlier than PCI. Thus, thrombolytic therapy remains an essential component of managing MI and should not be disregarded, especially in developing countries where PCI is not readily available and cannot be administered promptly. Fibrinolytics are medications that convert the proenzyme, plasminogen, into plasmin that degrades fibrin leading to the dissolution of clots [9]. The common fibrinolytics include streptokinase (SK) (first generation), alteplase (second generation), tenecteplase (TNK), and reteplase (third generation) [9]. The newer-generation fibrinolytics are produced to enhance the effectiveness and safety of alteplase [9]. TNK and SK are the two most commonly available/utilized fibrinolytics in managing MI [10]. However, according to ACCESS Registry, SK is the most frequently used in developing countries because it is less expensive [6], and 98% of MI patients in Thailand are treated with SK [11].

The major drawback of fibrinolytic therapy is recurrent infarction and bleeding especially intracranial hemorrhage (ICH) [12]. Elderly patients with low body weight and females are at increased risk of bleeding following thrombolytic treatment [12]. However, the rate and degree of adverse effects vary between the fibrinolytic agents. Controversy exists regarding the hierarchy of efficacy and safety of TNK vs STK [13,14]. Raja et al. reported that compared with TNK, SK has a more adverse reaction, lower rate of ST-segment resolution, less symptom resolution, a higher rate of bleeding, allergic reactions, and hypotension, and there must be one year gap before an individual can get another dose of SK [13]. On the other hand, Yazdi et al. reported similar safety and efficacy for TNK and SK in managing MI [14]. TNK is produced from alteplase by genetic engineering [15]. The three-point mutation of alteplase results in TNK, which is more fibrin specific and has a longer half-life [15]. Hence, it can be given as a single bolus. A meta-analysis conducted in 2017 reported that TNK has less bleeding risk than SK, alteplase, and reteplase [16]. Another study reported that TNK is cheaper, less difficult to administer, and has fewer bleeding complications than alteplase [17]. These properties of TNK (longer half-life, easier to administer, single bolus administration, and less bleeding risk) make it very suitable for use in developing countries where patients may have to travel for long hours to get PCI done. Another major challenge of thrombolytics is a dosing error and increased adverse effects in high-risk groups (insufficient dose for the obese and increased risk of bleeding for the elderly, females, and those with low body weight) [12]. The single-dose administration of TNK and its wide dosing range reduces dosing errors associated with other fibrinolytics [12]. Likewise, the Assessment of Safety and Efficacy of a New Thrombolytic (ASSENT 2) trial reported that the weight-optimized TNK, compared with alteplase, reduced the bleeding rate in the high-risk group [18]. Furthermore, those with high body weight who received weight-optimized TNK had reduced mortality compared with the rest [12]. SK has the benefit of fixed-dose, but its demerits of longer infusion time, antigenicity, decreased efficacy with repeated doses, and adverse reactions are significant concerns [19].

The result of several RCTs comparing the efficacy of thrombolytics in the Western world is also conflicting [20]. Although a meta-analysis of 40 RCTs has compared the efficacy and safety of fibrinolytics in managing MI, it also included trials conducted in the Western world before the era of dual antiplatelet therapy and anticoagulants, which is different from the current STEMI management guideline [10,21,22]. Another systematic review comparing SK and TNK reported similar efficacy and safety; however, it involved only four observational studies [23]. Several eligible studies were not included in the review. Method of practice and patient characteristics in the underdeveloped world vary from those of the Western world [20]; hence, these data may not be generalizable. The physician's choice of fibrinolytics is affected by the perceived superiority of TNK over SK and the high acquisition cost of TNK. Medicine cost is a major concern for patients in a developing country, and there is a dearth of data on the efficacy and safety of TNK vs SK. Hence, a systematic review of the effectiveness and safety of the two thrombolytics in developing countries is essential to ensure an optimal outcome for patients presenting with MI.

## Review

Aim

Many patients in the underdeveloped world do not have access to PCI. The efficacy of TNK vs SK in the developing world has not been reviewed systematically; therefore, this study aims to examine the available literature on the effectiveness and safety of TNK vs SK in managing MI in resource-poor nations.

Objectives

This study reviews available evidence on the effectiveness and safety of TNK vs SK in managing MI in resource-poor nations, determines the effectiveness and safety of TNK vs SK in managing MI in resource-poor nations, identifies possible adverse effects that preclude the use of TNK vs SK, and assess the quality of evidence on TNK vs SK, measure the outcome of TNK vs SK in the management of MI.

Methodology

The protocol and registration number of this study is CRD42022367107. The study is reported according to the Preferred Reporting Items for Systematic Reviews and Meta-analysis Extension (PRISMA) [24]. A meta-analysis seemed implausible because there was marked heterogenicity in the available studies; thus, a narrative synthesis was done according to Cochrane's synthesis without meta-analysis (SWIM) guidelines [25].

Inclusion criteria

Type of Study

Eligible studies assessed the efficacy and/or safety of TNK vs SK in managing MI. Studies that didn't mention TNK and/or STK in the title but mentioned fibrinolytics or thrombolytics were selected for full-text screening if a conclusion could not be reached on abstract screening. Similarly, studies that didn't include abstracts on the database but mentioned fibrinolytics on title screening were included for full-text screening. Likewise, studies were included for full-text screening, where a decision could not be reached based on title and abstract screening. No restriction was placed on the publication date, follow-up duration, and sample size. Besides systematic reviews, no restriction was placed on the study design. Studies conducted in developing countries were included. This was decided using the 2021 World Bank country classification by income level. Only countries in the lower- and middle-income levels were selected. Studies conducted in developed countries were also selected if they included lower- or middle-income populations.

Population

Studies focused on adults in developing countries were selected.

Intervention/Comparison

Eligible studies evaluated the efficacy and/or safety of TNK vs SK in treating MI. Studies comparing TNK vs SK and or other fibrinolytics were included; however, only the outcome of TNK vs SK was considered in the analysis.

Outcome

To be eligible, studies must report one of the primary safety or efficacy endpoint parameters. The principal safety endpoint entails major adverse events (major and minor bleeding, hemorrhagic stroke, mortality, arrhythmias, hypotension, shock, allergy, and anaphylaxis). The primary efficacy endpoint entails successful thrombolysis evidenced by a marked improvement in chest pain (symptom relief) or the resolution of ECG changes (complete ST-segment resolution and no ST-segment resolution). Other efficacy parameters include successful fibrinolysis, failed fibrinolysis, and myocardial reinfarction. Studies focused on cost-effectiveness were selected if any safety or efficacy endpoints were analyzed.

Exclusion criteria

Type of Study

Overlapping/repetitive studies, systematic reviews, non-English studies, conference abstracts, and studies conducted in high-income countries were excluded.

Population

Adults in the Western world and individuals less than 18 years were part of the exclusion criteria.

Intervention/Comparison

Studies focusing on other fibrinolytics besides TNK and SK were excluded. Similarly, studies conducted on only TNK or SK were not included for easy comparison.

Outcome

Studies that didn't report any safety or efficacy endpoints were excluded.

Search strategy

A comprehensive literature search was conducted on EMBASE, Cochrane Library, Web of Science, CINAHL, Scopus, Google Scholar, and Ovid version of MEDLINE databases using key search terms defined by the PICO (population, intervention, comparison, and outcome) framework. Reference lists of the eligible studies were manually searched. Additionally, the reference lists of excluded systematic reviews were searched, and eligible studies were selected. Boolean operators ("AND" "OR") were used as a search strategy. The search string included the following keywords: (tenecteplase OR TNK OR TNKase) AND (streptokinase OR SK OR STK) AND (AMI OR myocardial infarction OR heart infarction OR STEMI OR acute coronary syndrome). Only the first 38 pages (a total of 380 articles) of Google Scholar were screened. The articles were screened according to relevance so that the essential articles would be screened first.

Study selection

Studies that met inclusion/exclusion criteria were selected based on the titles and abstracts; the investigator then made a final selection based on the full-text article. The study selection was made in three stages of screening: title screening, abstract, and full-text. To ensure all relevant articles were included, study selection was conducted by two independent investigators. Inconsistency in opinion was resolved by discussion with a third reviewer.

Data extraction

All the relevant studies were merged into the Mendeley software (Elsevier, Amsterdam, Netherlands) and all duplicates were excluded. The extracted information was tabulated and included the author, publication date, design of studies, sample size, setting, follow-up duration, confounding factors, and outcome measures. See Tables 1-4 for study characteristics, baseline patient demographic/clinical characteristics, and outcome measures.

**Table 1:** Study characteristics SK: streptokinase, TNK: tenecteplase, RTP: reteplase, RCT: randomized controlled trial

Author/year/country	Sample size	Study design/setting	Follow-up duration	Outcome
Koh et al., 2022, Malaysia [28]	Total 698, SK 349, TNK 349	Single-center retrospective observational	30 days	Similar safety/efficacy, failed thrombolysis > TNK, hypotension and allergy > SK
Trerayapiwat et al., 2022, Thailand [11]	Total 25,907, SK 98%	Retrospective observational	In-hospital, 30 days, one year	TK more cost-effective
Shah et al., 2021, India [29]	Total 98, SK 54, TNK 44	Non-randomized quasi-experimental	In-hospital	TNK better reperfusion than STK
Neela et al., 2020, India [30]	Total 150, SK 50, TNK 50, RTP 50	Multicentre randomized study	In-hospital	TNK better safety and efficacy
Nikitha et al., 2020, India [31]	Total, 50, SK?, TNK?, RTP?	Prospective observational	In-hospital	Similar efficacy, TNK has fewer complications
Bawaskar et al., 2019, India [32]	Total 209, SK 162, TNK 47	Single-center prospective observational	30 days, one year	One year fatality > in STK but similar 30-day mortality
Chakka et al., India [13]	Total 70, SK 30, TNK 40	Prospective observational	In-hospital	TNK better safety and efficacy
Naini et al., 2019, India [33]	Total 20, SK 13, TNK 7	Prospective observational	In-hospital	TNK better ST-segment resolution and 2D echo result
Singh, 2019, India [34]	Total 150, SK 104, TNK 46	Prospective cohort	In-hospital	TNK faster rate of successful ST-segment resolution, SK higher allergic reaction
Aherrao et al., 2018, India [35]	Total 60, SK 30, TNK 30	Single-center randomized parallel study	Day 7, day 30, day 60	Similar safety and efficacy when given incorrect timelines
Yazdi et al., 2017, Iran [14]	Total 142, SK 88, TNK 54	Cross-sectional study	In-hospital	Similar safety, efficacy, and complication
Deshani et al., 2016, India [36]	Total 60, SK 30, TNK 30	Retrospective observational	In-hospital	TNK better efficacy and safety
Xavier et al., 2016, India [37]	Total 90, SK 30, TNK 30, RTP 30	Prospective observational	In-hospital	TNK better efficacy and safety
Panduruga et al., 2012, Yemen and five Middle Eastern countries [38]	SK 674, TNK 164, RTP 700	Prospective multicenter observational	In-hospital, one month, one year	TNK has lower one-month and one-year mortality
Al-Zakwani et al., 2012, Yemen and five Middle Eastern countries [39]	SK 454, TNK 532	Prospective multicenter observational	In-hospital	TNK lower all-cause mortality
Pulluri et al., 2014, India [40]	Total 90, SK 30, TNK 30, RTP 30	Retrospective observational	In-hospital	TNK is safer and more effective than SK
Giraldez et al., 2009, 48 countries [41]	Total 18,366, SK 2,083	RCT	In-hospital day 30	STK + enoxaparin similar safety and efficacy

**Table 2:** Baseline patient’s demographics and clinical characteristics SK: streptokinase, TNK: tenecteplase, NR: not recorded, STP: symptom-to-treatment time, DTN-: door-to-needle time, Rx: treatment, HGS: high grace score, Inf: inferior, Ant: anterior, DM: diabetes mellitus, HF: heart failure, HTN: hypertension, SBP: systolic blood pressure, MI: myocardial infarction, TIMI: thrombolysis in myocardial infarction

Study	Age (mean)	M sex (%)	DM (%)	HF (%)	HTN (%)	SBP (mean)	Smoker (%)	Onset Rx time (hr)/STP (min)	Door-to-needle time (min)	Killip class >11 (%)	MI location/type (%)	TIMI >30/HGS (%)	
Koh et al., 2022 [28]	SK 52.9, TNK 53.6	SK 89.7, TNK 88.8	SK 37.8, TNK 38.8	SK 1.7, TNK 1.7	SK 44.7, TNK 45.3	SK 129.3, TNK 128.2	SK 68.2, TNK 68.5	<4 hrs, SK 67.3%, TNK 67%	NR	SK 25, TNK 25.1	Ant SK 55, TNK 59	NR
Trerayapiwat et al., 2022 [11]	NR	NR	NR	NR	NR	NR	NR	NR	NR	NR	NR	NR
Shah et al., 2021 [29]	<65-74, 65-74 -14 >74-10	79	NR	NR	NR	NR	NR	≤2 hrs 49, >2 hrs 49	NR	NR	Ant 51	NR
Neela et al., 2020 [30]	>60, SK 46, TNK 22	SK 68, TNK 82	NR	NR	42.6	NR	42	NR	NR	NR	NR	NR
Nikitha et al., 2020 [31]	NR	NR	NR	NR	NR	NR	NR	NR	NR	NR	NR	NR
Chakka et al., 2019 [13]	>45, 77.14	82.5	NR	NR	NR	NR	NR	NR	NR	NR	Ant 55.7, Inf 44.2	NR
Bawaskar et al., 2019 [32]	>70, SK 22.4, TNK 6.3	SK 79.6, TNK 89.1	SK 29.1, TNK 44.68	NR	SK 62.9, TNK 80.85	NR	SK 44.44, TNK 42.53	STP >3 hrs, SK 40.74%, TNK 12.76%	>60, SK 20.3, TNK 32.76	NR	Ant/inf SK 56.7/49.2, TNK 54.3/41.3	NR
Naini et al., 2019 [33]	NR	NR	NR	NR	NR	NR	NR	NR	NR	NR	NR	NR
Singh, 2019 [34]	SK 49.5, TNK 50.4	SK 75, TNK 76.1	SK 29.8, TNK 19.6	NR	SK 56.7, TNK 63	SK 102, TNK 104	SK 33.33, TNK 39.1	NR	NR	NR	Ant/Inf SK 9/39.4, TNK 50/41.3	NR
Aherrao et al., 2018 [35]	NR	NR	NR	NR	NR	NR	NR	NR	NR	NR	NR	NR
Yazdi et al., 2017 [14]	SK 52.07, TNK 52.76	SK 56.8, TNK 55.6	SK 42, TNK 33	NR	NR	NR	NR	NR	SK 42, TNK 45	NR	Ant/extensive SK 18.2/15.9, TNK 13/27.8	NR
Deshani et al., 2016 [36]	NR	NR	NR	NR	NR	NR	NR	NR	NR	NR	NR	NR
Xavier et al., 2016 [37]	NR	SK 70, TNK 56.7	NR	NR	NR	NR	NR	NR	NR	NR	NR	NR
Pulluri et al., 2014 [40]	NR	SK 76.6, TNK 90	DM+HTN SK 70, TNK 63.3	NR	NR	NR	SK 33.3, TNK 20	NR	NR	NR	NR	NR
Panduruga et al., 2012 [38]	SK 53, TNK 49	SK 89, TNK 92	SK 28, TNK 31	SK 0.6, TNK 2.4	SK 33, TNK 29	NR	SK 50, TNK 48	STP SK 190 min, TNK 170 min	DTN 40, 34	SK 4.9, TNK 18	Extensive/Ant SK 22/34, TNK 16/37	HGS SK 15, TNK 25
Al-Zakwani et al., 2012 [39]	SK 55, TNK 50	SK 85, TNK 92	SK 27, TNK 36	NR	SK 25, TNK 33	SK 130, TNK 139	SK 53, TNK 59	STP SK 165 min, TNK 120 min	SK 50, TNK 38	SK 4.4, TNK 4.6	NR	HGS SK 22, TNK 10
Giraldez et al., 2009 [41]	SK 60, TNK 59	SK 74.8, TNK 77.4	SK 17.1, TNK 14.7	NR	SK 42.3, TNK 45	NR	NR	Sk 3.2 TNK 3.1	NR	SK 10.1, TNK 11.5	Ant SK 35.7, TNK 45.6	SK 36.1, TNK 35.4

**Table 3:** CASP cohort checklist ✓: yes, X: no, ?: can't tell

Criteria	Koh et al., 2022 [28]	Trerayapiwat et al., 2022 [11]	Nikitha et al., 2020 [31]	Bawaskar et al., 2019 [32]	Chakka et al., 2019 [13]	Naini et al., 2019 [33]	Singh, 2019 [34]	Yazdi et al., 2017 [14]	Deshani et al., 2016 [36]	Xavier et all., 2016 [37]	Panduranga et al., 2012 [38]	Al-Zakwani et al., 2012 [39]	Pelluri et al., 2014 [40]	Giraldez et al., 2009 [41]
Focused	✓	✓	✓	✓	✓	✓	✓	✓	✓	✓	✓	✓	✓	✓
Acceptable recruitment	✓	✓	✓	✓	✓	✓	✓	✓	✓	✓	✓	✓	✓	✓
Accurate measurement of exposure	✓	✓	✓	✓	✓	✓	✓	✓	✓	✓	✓	✓	✓	✓
Identification of confounding factors	✓	✓	X	✓	✓	X	✓	✓	X	X	✓	✓	?	✓
Consideration of confounding factors	✓	✓	X	✓	✓	X	✓	✓	X	X	✓	✓	?	✓
Adequate duration of follow up	?	✓	X	✓	X	X	X	X	X	X	✓	X	?	?
Result	✓	✓	✓	✓	✓	✓	✓	✓	✓	✓	✓	✓	✓	✓
Precise result	✓	✓	✓	✓	✓	✓	✓	✓	✓	✓	✓	✓	✓	✓
Reliable result	✓	✓	?	?	✓	?	✓	✓	?	?	✓	✓	✓	✓
Generalizable	✓	✓	?	✓	?	?	✓	✓	?	?	✓	✓	✓	✓
Correspond with available evidence	✓	✓	✓	✓	✓	✓	✓	✓	✓	✓	✓	✓	✓	✓
Practical implication	✓	✓	✓	✓	✓	✓	✓	✓	✓	✓	✓	✓	✓	✓

**Table 4:** Outcome measures SK: streptokinase, TNK: tenecteplase, NR: not recorded, ↑SK: value not recorded, but it was mentioned that SK had a higher value in those circumstances, Tach: tachycardia, VT: ventricular tachycardia, VF: ventricular fibrillation, Fib: fibrillation, TIMI: thrombolysis in myocardial infarction

Outcome measures	Koh et al., 2022 [28]	Trerayapiwat et al., 2022 [11]	Neela et al., 2020 [30]	Nikitha et al., 2020 [31]	Bawaskar et al., 2019 [32]	Sekhar et al., 2019 [13]	Naini et al., 2019 [33]	Singh, 2019 [34]	Aherrao et al., 2018 [35]	Yazdi et al., 2017 [14]	Deshani et al., 2016 [36]	Xavier et al., 2016 [37]	Panduruga et al., 2012 [38]	Al-Zakwani et al., 2012 [39]	Giraldez et al., 2009 [41]	Shah et al., 2021 [29]	Pulluri et al., 2014 [40]
In-hospital mortality (%)	SK 10, TNK 12.9	NR	NR	NR	SK 13.3 TNK 6.68	NR	NR	NR	NR	Death SK 11.4, TNK 14.8	NR	SK 3U, TNK 3	SK 4.6, TNK 6.7	↑SK	Death SK 7.5, TNK 6.7	NR	SK 3.33, TNK 3.33
30-day mortality (%)	SK 11.2, TNK 13.2	↑SK	NR	NR	NR	NR	NR	NR	SK 0, TNK 0	NR	NR	NR	SK 4.2, TNK 0.8	NR	NR	NR	NR
Adverse reaction (%)	NR	NR	NR	NR	NR	SK 60, TNK 15	NR	SK 24, TNK 15.5	NR	NR	NR	NR	NR	NR	NR	NR	NR
One-year mortality (%)	NR	↑SK	NR	NR	SK 7.4 TNK 0	NR	NR	NR	NR	NR	NR	NR	SK 3.4, TNK 0	NR	NR	NR	NR
Hemorrhagic stroke (%)	SK 0.9 TNK 0.3	NR	NR	NR	NR	NR	NR	SK 1.9, TNK 2.2	NR	NR	SK 0, TNK 0	NR	NR	NR	NR	NR	SK 0, TNK 3.33
Major bleeding (%)	SK 1.1 TNK 0.9	↑SK	SK 10, TNK 4	SK 14.3, TNK 11.1	SK 9.25 TNK 6.38	↑SK	NR	NR	SK 0, TNK 0	SK 5.7, TNK 11.1	SK 0, TNK 0	SK 0, TNK 0	NR	↑SK	SK 2.4, TNK 2.0	NR	SK 0, TNK 0
Minor bleeding (%)	SK 3.4, TNK 0.9	NR	NR	NR	NR	NR	NR	Moderate, SK 17.3, TNK- 13	NR	SK 22.7, TNK 24.1	SK 0, TNK 0	NR	NR	NR	SK 3.8, TNK 2.2	NR	NR
Successful fibrinolysis/TIMI 3 (%)	SK 93.1, TNK 88.5	NR	NR	NR	SK 31.92 TNK 30.50	NR	NR	NR	TIMI SK 57, TNK 60	NR	NR	NR	NR	NR	NR	SK 46.4 TNK 54.3	NR
Failed fibrinolysis (%)	SK 5.7, TNK 10.6	NR	NR	NR	SK 4.3 TNK 8.5	NR	NR	NR	NR	NR	NR	NR	NR	NR	NR	NR	NR
Reinfarction (%)	SK 5.7, TNK 5.7	NR	NR	NR	SK 2.5 TNK 6.38	NR	NR	SK 26, TNK 21.7	NR	NR	NR	NR	NR	NR	NR	NR	SK 0, TNK 3.33
Hypotension (%)	SK 38, TNK 14.3	NR	SK 6, TNK 4	SK 0, TNK 18.5	SK 20.98 TNK 17.02	↑SK	NR	Shock SK 8.7, TNK 8.7	NR	NR	Shock SK 16.7, TNK 10	SK 16.6, TNK 3	NR	↑SK	NR	NR	SK 3.33, TNK 0
Bradycardia (%)	SK 10.3, TNK 6.9	NR	NR	NR	SK 19.7 TNK 19.14	NR	NR	NR	NR	NR	NR	NR	NR	NR	NR	NR	NR
Arrhythmias/tachycardia (%)	NR	NR	NR	NR	Tach SK 9.25, TNK 2.1	↑SK	NR	Arryth SK 10.6, TNK 6.5	NR	NR	NR	Arryth SK 3, TNK 0	NR	NR	NR	NR	Arryth SK 3.33, TNK 3.33
Ventricular fibrillation/ tachycardia (%)	SK 15.2, TNK 17.5	NR	NR	Tach SK 0 TNK 3.5	VT/VF SK 3.7/3.08, TNK 6.3/10.6	NR	NR	NR	NR	NR	SK 23.35, TNK 20	NR	NR	NR	NR	NR	NR
Atrial fibrillation/tachycardia (%)	SK 3.2, TNK 4	NR	NR	NR	Fib SK 1.23, TNK 0	NR	NR	NR	NR	NR	NR	NR	NR	NR	NR	NR	NR
Anaphylaxis (%)	SK 0.9, TNK 0.6	NR	SK 2, TNK 0	NR	NR	NR	NR	NR	NR	NR	SK 0, TNK 0	NR	NR	NR	NR	NR	NR
Allergy (%)	SK 2.9, TNK 0.6	NR	NR	NR	NR	↑SK	NR	SK 4.8, TNK 0	NR	NR	NR	SK 0, TNK 0	NR	NR	NR	NR	SK 3.33, TNK 0
Ejection fraction >40%	NR	NR	NR	NR	NR	NR	NR	NR	NR	NR	SK 73.3, TNK 63.3	NR	NR	NR	NR	NR	NR
Complete ST-segment resolution (%)	NR	NR	>50%, SK 52.9, TNK 70	SK 57.2 TNK 59.3	NR	>50% SK 46.6, TNK 77.5	SK 23, TNK 43	SK 50, TNK 67.39	SK 73.3, TNK 76.6	Extent SK 0.8, TNK 1.02	>50% SK 80, TNK 90	>50% SK 83, TNK 83	NR	NR	NR	NR	>50% SK 90, TNK 86.66
No ST-segment resolution (%)	NR	NR	SK 34, TNK 20	SK 28.5 TNK 37	SK 4.32, TNK 8.51	NR	SK 31, TNK 14	SK 19.23, TNK 8.7	NR	NR	NR	NR	NR	NR	NR	NR	NR
Symptom relief (%)	NR	NR	SK 94, TNK 98	NR	NR	SK 49.9, TNK 50	NR	NR	NR	NR	>50 SK 76.6, TNK 86.6	SK 86, TNK 90	NR	NR	NR	NR	SK 93.33, TNK 83.33

Critical appraisal/quality improvement

Each RCT was assessed for risk of bias using the Cochrane tool Risk of Bias 2 (ROB2; Cochrane, London, England) for assessing the risk of bias [26]. They were evaluated for risk of bias utilizing the components of ROB2, which include random sequence generation, allocation concealment, blinding of participants and personnel, blinding of outcome assessment, incomplete outcome data, and selective reporting. Each study's protocol was sought in the clinical trial register to assess the risk of bias due to selective reporting. However, only the Enoxaparin and Thrombolysis Reperfusion for Acute Myocardial Infarction Treatment (EXTRACT TIMI-25) trial protocol was retrieved, and all outcomes were reported (study by Giraldez et al. is a subanalysis of EXTRACT TIMI-25). EXTRACT TIMI-25 was reported as having a low risk of bias because of double blinding; efforts at allocation concealment were made using a computerized random number generation sequence. The study by Neha et al. and Neela et al. were graded high risk of bias because there was no information on allocation concealment, random sequence generation, blinding, and reporting.

Quality assessment

The quality of the RCTs and non-quasi-experimental studies was assessed using the Grading of Recommendations Assessment, Development, and Evaluation (GRADE; Cochrane, London, England) tool [25] against the risk of bias, inconsistency, indirectness, imprecision, publication bias, and upgrading factors. The quality of the observational studies was assessed using the Critical Appraisal Skills Programme (CASP) cohort study checklist [27]. Based on bias assessment, the studies were grouped as low, medium, or high. See Table 4 for details.

Data synthesis/analysis

**Figure 1:**
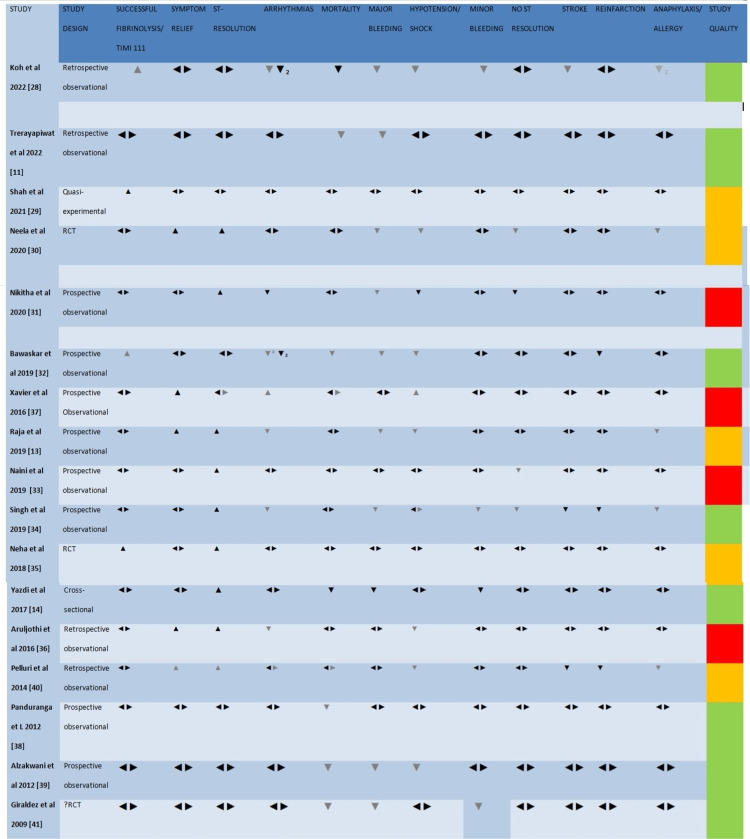
Effect direction plot The upward arrow ▲ signifies positive health impact, the downward arrow ▼indicates negative health impact, the multi-colored bi-directional arrow ◄► represents a similar effect between the two agents, and the darker bi-directional arrow ◄► suggests no recorded event by the two agents (not included in the analysis). The darker arrow ▲ represents TNK, while the lighter one ▲ represents SK. Final sample size (individuals in the intervention group): the big arrow ▲ represents a sample size greater than 300, the medium arrow ▲ represents a sample size of 50-300, and the small arrow ▲ represents a sample size less than 50. The row color donates the quality of the study: green denotes a low risk of bias, amber denotes some concerns regarding the quality, and red denotes a high risk of bias.

Only studies that met the eligibility criteria and passed the three-stage selection process were synthesized. After a comprehensive evaluation of the eligible studies, a deduction of heterogeneity was made regarding the outcome measures; hence, data were analyzed using a formal narrative synthesis. It was adopted following the Cochrane guideline for SWIM [25]. An effect direction plot was used to present the result of the narrative data synthesis (see Figure 1). An upward arrow was used for the intervention with a positive conclusion. A positive conclusion entails successful thrombolysis, symptom relief, and ST-segment resolution. A downward pointing was used for the intervention with negative conclusions. A negative conclusion entails failed thrombolysis, no ST-segment resolution, mortality, stroke, bleeding, hypotension/shock, reinfarction, and arrhythmias. A multi-colored bidirectional arrow was used for studies with similar outcomes between TNK and SK. In contrast, a dark bi-directional arrow was used for studies that recorded no event in the two arms (they were not included in the synthesis). A vote counting was done based on the direction of the effect. The direction of the effect is reported if ≥70% of the outcome reports the same direction. If less than 70%, the direction of the effect is reported; however, a conclusion of conflicting findings was made [25]. See the result section for details of the effect direction plot in Figure 1 and the result of the vote counting.

Result

**Figure 2:**
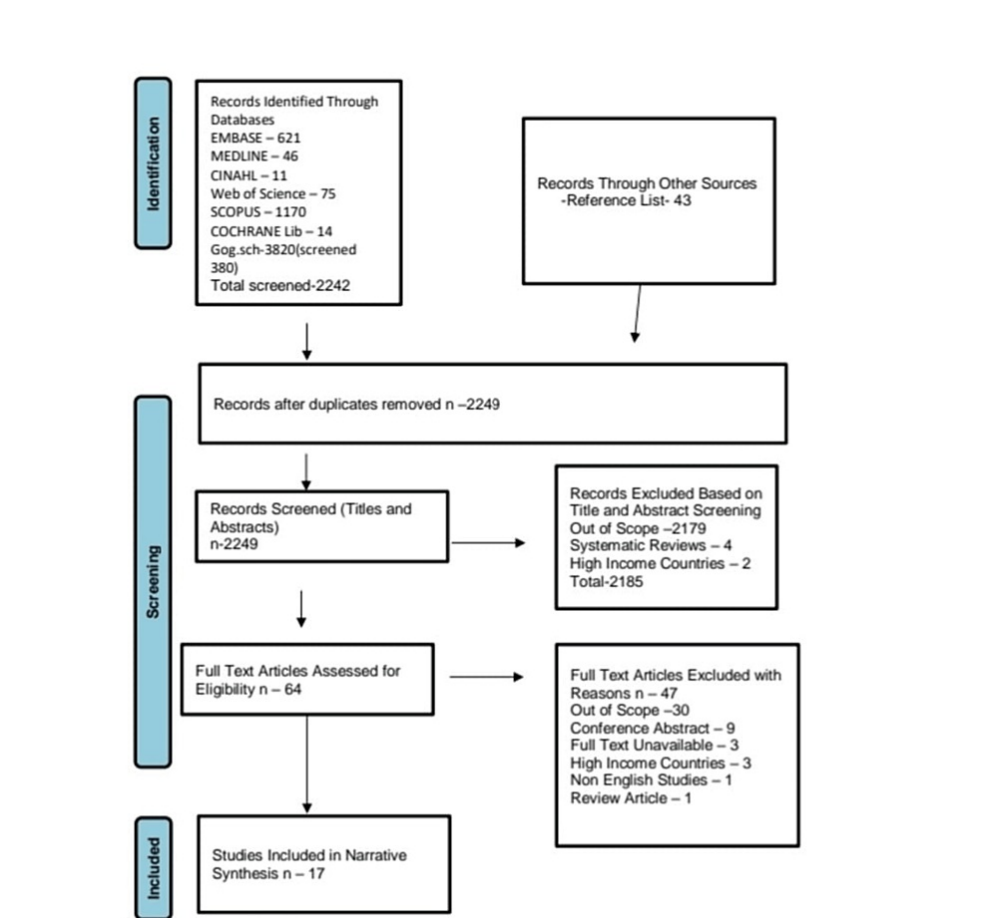
PRISMA diagram

The study selection process is shown in the PRISMA diagram in Figure 2.

Of the 2284 references retrieved from the databases, only 17 studies [11,13,14,28-41], with a cumulative total of 36,957 participants, met the inclusion criteria and were selected for final analysis. The study characteristics (date, country, size, design, and outcome) are shown in Table 1. Eight are prospective observational studies [13,31-34,37-39], four are retrospective observational [11,28,36,40], one is cross-sectional [14], one is non-randomized quasi-experimental [29], and three are RCTs [30,35,41]. The study by Giraldez et al. is a subanalysis of an RCT (EXTRACT TIMI-25). The selection of anticoagulants (unfractionated heparin vs enoxaparin) was randomized, while the fibrinolytics were selected at the physician's discretion; thus, the quality was assessed as an RCT and an observational study [41]. Ten studies were conducted in India [13,29-37,40], while the rest were conducted in Malaysia [28], Thailand [11], Iran [14], Africa/Asia, and Yemen. Studies by Panduranga et al. and Alzakwani et al. were conducted in Yemen and five other high-income countries [38,39]. Giraldez et al. conducted their study in 48 countries, including some developing countries in Africa and Asia [41]. All articles were published between 2009 and 2022.

The sample size ranges from 20 to 25,907. TNK arm involves 7,226 (19.5%) participants, while the SK arm involves 29,731 (80.5%) participants. The sample sizes of five studies [11,28,38,39,41] are noticeably large: 698, 25,907, 7,510, 986, and 838, respectively. Of four studies [14,30,32,34] are in-between, 142, 150, 209, and 150, while eight studies [13,29,31,33,35,36,37,40] have apparent small sample sizes (considering that this number is shared between TNK and SK), 50, 70, 98, 60, 60, 60, 60, and 20 respectively. Most studies have more participants in the SK arm (25,907/518, 162/47, 88/54, 674/164, 88/54, 13/7, 104/46, 54/44); however, six studies [28,30,35-37,40] have an equal number of participants in TNK and SK arm, while three studies [13,39,41] have more participants in TNK arm: 40/30, 532/454, and 5427/2083. Only 2% of participants in the study by Trerayapiwat et al. used TNK. They used data from the national register from 2012 to 2019 and involved 25,907 cohorts. To avoid selection bias, data from the national record was not used in the TNK arm [11]. Instead, the probabilities of the event in the TNK arm were calculated utilizing the risk ratio of events in the TNK arm compared to SK, which was derived from a 2017 meta-analysis comparing the efficacy/safety of four fibrinolytics in MI patients [16].

The baseline patients' demographics are illustrated in Table 2. For studies that used mean age, there is not much difference between SK and TNK groups. Neela et al. and Bawaskar et al. recorded more elderly participants in the SK group [30,32]. All the studies have predominantly male cohorts in both arms except that by Yazdi et al., which has 55.6% and 56.8% of males in the TNK and SK groups, respectively [14]. Three studies combined patients' demographics and clinical characteristics [29,36,37]. The number of participants in TNK and SK was not indicated. Naini et al. made no mention of patients' clinical characteristics or demographics [33].

Quality assessment

The study by Giraldez et al. was assessed as an RCT and an observational study (it's a sub-analysis of the EXTRACT TIMI-25 trial where patients were randomized to receive anticoagulants while the use of fibrinolytics was at the discretion of the physician). It was graded high quality because, as an RCT, there is consistency, directedness, precision, and low risk of bias. As an observational study, multivariate models were utilized to adjust for imbalances between groups, and a propensity score was used to adjust for selection bias [41]. Studies by Neha et al. and Neela et al. have a high risk of bias and small sample size; however, they were graded as moderate quality because they are RCTs [30,35]. Shah et al.'s quasi-experimental study was graded as high quality using Cochrane's GRADE tool [29]. Using the CASP study checklist (Table 3), nine observational studies [11,13,14,28, 31,32-34,38,40] were graded as high quality because measures were taken to adjust for the confounding factors. They were equally graded as high quality with the GRADE criteria. Four studies [31,33,36,37] were graded as low-quality due to the non-identification of confounding factors and the small sample size.

Outcome measures/effect direction plot

The outcome measures of this study are portrayed in an effect direction plot (Figures 2, 4). The effect direction plot effectively visually represents the characteristics/outcome of studies in SWIM. It also represents evidence of a positive, negative, or no change in effect [42]. This study compares the efficacy and safety of two fibrinolytics (TNK and SK). Each outcome measure is represented on the plot by the fibrinolytic with a higher value without considering the P-value/statistical significance [42]. The efficacy endpoint was assessed by symptom relief, ST-segment resolution, successful fibrinolysis, no ST-segment resolution, failed fibrinolysis, and reinfarction. On the other hand, the safety endpoint was recorded as arrhythmia, mortality/death, bleeding, hypotension/shock, stroke, and anaphylaxis/allergy.

Regarding efficacy endpoint, five studies reported symptom relief [13,30,36,37,40]. Complete ST-segment resolution was reported by 10 studies [13,14,30,31,33,35-38,40]. Four studies reported successful fibrinolysis/reperfusion or TIMI 111 [28,29,32,35]. Five studies reported no ST-segment resolution [14,30,31,33,34]. Two studies reported failed fibrinolysis [28,32], while reinfarction was reported by four studies [28,32,34,40].

For the safety endpoints, eight studies reported arrhythmia [13,28,31,32,34,36,37,40]. Ten studies reported mortality [11,14,28,32,35,37-41]; however, one study recorded no event; therefore, it was excluded from the synthesis. Eight studies reported in-hospital mortality [14,28,32,37-40]; three reported 30-day mortality [11,28,38] and one-year mortality [38]. Two studies reported adverse reactions [13,32]. Fourteen studies reported bleeding [11,13,14,28,30-32,34-37,39-41]; however, four studies recorded no bleeding episodes in both arms, possibly because they have a small sample size [35-37,40]. Nine studies reported hypotension/shock [13,28,30-32,34,36,37,40]. Four studies reported stroke, but one recorded no event [28,34,36,40]. Anaphylaxis/allergy was reported by seven studies, but two recorded no event [13,28,33,34,36,37,40].

Vote counting

The vote counting for the efficacy parameters is as follows: symptom relief (TNK 80%, SK 20%), complete ST-resolution (TNK 80%, SK 10%, same effect 10%), successful fibrinolysis (TNK 50%, SK 50%), no ST-resolution (TNK 40%, SK 60%), failed fibrinolysis (not synthesized), and RE infarction (TNK 50%, SK 25%, same effect 25%).

The vote counting for the safety parameters is as follows: arrhythmias TNK (28.6%, SK 64.3%, same effect 7.1%), mortality (TNK 28.6%, SK 57.1%, similar effect 14.3%), in-hospital mortality (TNK 37.5%, SK 37.5%, similar effect 25%), 30-35 day mortality (TNK 33.3%, SK 66.7%), one-year mortality (TNK 0%, SK 100%), adverse reactions (not synthesized), minor bleeding (TK 25%, SK 75%), major bleeding (TNK 11.1%, SK 88.9%), hypotension/shock (TNK 11.1%, SK 77.8%, similar effect 11.1%), stroke (TNK 66.7%, SK 33.3%), and anaphylaxis/allergy (TNK 0%, SK 100%).

According to Boon and Thomson, 2021, when analyzing the result of the vote counting of an effect direction plot, ≥70% of the direction of an outcome measure has to report in a similar direction for the effect to be reported [42]. If it's less than 70%, the direction of the effect with the majority is reported; however, a conclusion of conflicting findings will be made [42]. Based on this, regarding efficacy parameters, TNK is better than SK in terms of symptom relief (on average, TNK causes symptom relief in 85.1% of cases vs 76.8% by SK. Its direction of effect also crossed the 70% threshold on the plot, 80% vs 20%). In terms of complete ST-segment resolution, TNK also shows superiority over SK (80% vs 10% on the plot, on average, TNK vs SK is 71.3% vs 59.13%). Both fibrinolytics show similar efficacy regarding successful fibrinolysis/TIMI 111 flow (50% vs 50 on the plot and 58.3 vs 57.1% on average). There is a conflicting finding regarding reinfarction (50% vs 25% on the plot, and 9.3% vs 8.6% on the average) and no ST-segment resolution (60% vs 40% on the plot and 23.41 vs 17.64 on the average). Though TNK appears to have a higher risk of reinfarction, and the SK group recorded more no-ST-segment resolution, they didn't meet the 70% threshold. Failed fibrinolysis was not analyzed because it was reported by only two studies, though it was worse in the TNK group.

Regarding safety endpoints, SK, compared to TNK, was associated with a higher rate of major bleeding (88.9% vs 11.1% on the plot and 7.1% vs 5.9% on average), minor bleeding (75% vs 25% on the plot and 11.8% vs 10.1%), hypotension (77.8% vs 11.11% on the plot and 13.8% vs 7.2% on the average), and anaphylaxis/allergy (100% vs 0% on the plot and 2.32% vs 0.6% on the average). TNK is associated with more episodes of hemorrhagic stroke (66.7% vs 33.3% on the plot and 1.94% vs 0.93% on the average); however, it is below the landmark; hence, a conclusion of conflicting evidence was made. On the SK arm, higher rate of arrhythmia (64.3% vs 28.6% on the plot and 16.5% vs 8.1% on the average) and mortality (57.1% vs 28.6% on the plot and 8.48% vs 7.23% on the average) was recorded. Still, a conclusion of conflicting evidence was made for the same reason. On sub-analysis of mortality, a higher risk of one-year mortality was recorded in the SK arm (100% vs 0% on the plot and 10.8% vs 0% on the average). In-hospital mortality was similar among the agents (37.5% vs 37.5% on the plot and 7.7% vs 7.6% on the average), while a conclusion of conflicting findings was made for 30-day mortality.

Discussion

Brief History of Fibrinolytics

Carl Weigert William postulated in 1980 that the major cause of MI is plaque rupture which leads to intracoronary thrombosis and blockage of blood in the distal coronary arteries [43]. Treatment of MI was exclusively by palliative measures until 1950 [43]. In the current era, managing MI entails urgent blood flow restoration [43]. The first fibrinolytic agent to achieve this is SK [43]. SK was introduced in 1933 by Associate Professor of Medicine William Tillet after discovering that streptococcus agglutinated in test tubes filled with plasma but not with serum [44]. He inferred that the agglutination of the organism resulted from a substance in the plasma, which is absent in the serum. He concluded that streptococci produce a thrombolytic agent that lysis blood clots in plasma. This led to the invention of the fibrinolytic agent SK [44]. Its application in cardiology took over 30 years to be fully recognized by physicians [19]. SK was used for treating many clinical conditions until 1960 when Boucek and Murphy first used it to treat patients with MI [45]. In 1976, Chazou et al. suggested that intracoronary administration of SK is more effective than systemic administration [46]. However, subsequent studies contraindicated their finding as no route of administration was found to be better [47]. In 1979, after many preliminary small sample studies, a trial conducted with 2388 cohorts concluded that combined treatment of SK with heparin reduced six months mortality following MI compared to standard heparin infusion [48]. Multiple trials have demonstrated the effectiveness of SK in reducing mortality associated with MI [19,48]. In 1985, an Italian Group for the Study of Streptokinase in Myocardial Infarction (Gruppo Italiano per lo Studio Della Streptochinasi nell'Infarto Miocardico (GISSI)) trial was conducted with over 11,000 patients, and it reported that intravenous fibrinolysis with SK decreased 12 months mortality after MI [49]. French et al. established that TIMI 3 flow, a marker of successful fibrinolysis and prognosticator of mortality [50], was improved in 33% of patients following the administration of SK [51]. International Studies of Infarct Survival (ISIS) 2 trial is a large RCT that has also demonstrated the efficacy of SK, leading to its universal use [52]. Fibrinolytics are associated with several adverse effects, such as hemorrhagic stroke and bleeding [53]. SK shouldn't be given to a patient in less than a space of one year [19].

Adverse reactions like allergy, anaphylaxis, and hypotension linked with SK led to the production of newer fibrinolytics with fewer adverse reactions and easy administration [54-58]. The newer fibrinolytic agents produced include recombinant tissue plasminogen activators (t-PA), alteplase, reteplase, and TNK [19]. They are less antigenic and, hence, has a lower risk of allergy and anaphylaxis and are more fibrin specific. Alteplase is administered only by intravenous route; its half-life is short [19]. Reteplase has a longer half-life and can be given as a bolus to MI patients [19]. TNK is a third-generation fibrinolytic, the most fibrin specific lytic, and more resistant to endogenous t-PA inhibitors than other agents. It is given as a bolus over 5-10 seconds [59,60]. TNK has a longer half-life and has proven beneficial based on the result of the thrombolysis in myocardial infarction (TIMI) 10A, ASSENT 1, and ASSENT 2 trials [61-63]. However, these features of newer-generation thrombolytics do not translate to better efficacy or safety. For instance, the new fibrinolytic agent lanoteplase was withdrawn because it was associated with a high rate of hemorrhagic stroke [64,65]. The following contra-indication precludes the use of fibrinolytic agents: recent internal hemorrhage, intracerebral hemorrhage, ischemic stroke within the last six months, pregnancy, uncontrolled high blood pressure, major surgery, recent trauma with resuscitation, etc. [66].

Despite the rising rate of MI and the well-documented survival benefit of primary PCI in its management, several low- and middle-income countries still have difficulties providing PCI on time [67-69]. Fibrinolytics, therefore, remains an indispensable alternative therapy in this circumstance. Fibrin-specific thrombolytics (e.g., TNK) are favored over non-fibrin-specific thrombolytics (e.g., STK) due to documented evidence of better effectiveness and safety; hence, several international guidelines advocate the use of fibrin-specific agents [3,70,71]. The high acquisition cost of fibrin-specific agents has made it difficult for people in resource-poor nations to afford them; consequently, SK is the most commonly used worldwide [6,11].

Comparison of Study Outcome With Available Evidence/Inferences

This is the first systematic review comparing the outcome of TNK vs SK in low- and middle-income countries. Several guidelines in the Western world have indeed advocated for fibrin-specific agents over SK [70,71]. Still, studies conducted in the Western world may not be generalizable due to diversity in physique, disease, and treatment outcome [72]. Additionally, significant differences exist in the developmental stages of high-income and low- and middle-income countries. This disparity can cause variability in the performance of fibrinolytics in both regions [72,73]. Due to the shortage of data and conflicting outcomes of several studies regarding the safety and efficacy of TNK vs SK, this systematic review was carried out to establish which thrombolytic is superior in managing MI in the emerging world. Most physicians worry about vital safety parameters when administering fibrinolytics to patients, including bleeding, hemorrhagic stroke, and mortality.

Unfortunately, in this review, only four studies reported hemorrhagic stroke [28,34,36,40]. One of the studies reported no incidence of stroke in both arms, probably due to the small number of cohorts (30 in each component) recruited for their research [36]. This study's rate of hemorrhagic stroke between TNK and SK is inconclusive. This controversy is also comparable with the findings of other literature. Two retrospective observational studies carried out in Hong Kong reported two different conclusions for hemorrhagic stroke between TNK and SK; McCormick et al. reported a higher rate of hemorrhagic stroke with TNK than with SK [74], while Chau et al. reported a similar stroke rate of [75]. The available systematic reviews also reported conflicting findings. A 2003 meta-analysis of 14 RCTs reported a higher risk of stroke with TNK than SK [23], while a 2017 meta-analysis of 40 RCTs reported the opposite; they suggested that TNK has a lower stroke risk than SK [16]. On the other hand, a 2016 systematic review that analyzed data in the context of high-income countries reported no difference in stroke rate between the two agents [76]. Notably, these RCTs analyzed by the systematic reviews were carried out before the era of dual antiplatelet therapy and anticoagulants. It is also critically vital to unravel this perplexity concerning the risk of hemorrhagic stroke between fibrinolytics, as studies have shown that hemorrhagic strokes following the administration of fibrinolytics are usually fatal. Koh et al. reported that all cases of stroke following the administration of fibrinolytics resulted in death [28]. To unravel this uncertainty, large, multicenter RCTs are needed in this era of dual antiplatelet therapy and anticoagulants.

This study concluded that TNK has a lower risk of major and minor bleeding than SK. This finding corresponds with the conclusions of two meta-analyses of RCTs on the safety of four fibrinolytics (TNK, SK, alteplase, and reteplase) carried out in 2003 and 2017 [16,23]. They established that TNK has a lesser risk of bleeding than other thrombolytic agents. However, a meta-analysis of four observational studies of direct comparison of SK and TNK reported a similar risk of bleeding between the two agents [77]. Some high-income countries also reached varying conclusions, although the type of bleeding was not specified; in their study conducted in Lithuania, Serpytis et al. reported no bleeding risk in both arms [66]. In contrast, Chau et al. reported a similar bleeding risk in their study in Hong Kong [75].

As regards mortality, the evidence is conflicting. However, more studies reported a higher risk of mortality with SK than with TNK. On a sub-analysis of one-year mortality, SK was found to have a higher risk of long-term mortality than TNK. In-hospital mortality was similar between SK and TNK, while the result of 30-35-day mortality is conflicting. This study's long-term mortality result agrees with the Global Utilization of Streptokinase and Tissue Plasminogen Activator for Occluded Coronary Arteries (GUSTO) trial's findings, which reported similar 30-day mortality between SK and other fibrinolytic agents but significantly higher one-year mortality in the SK arm [58]. Similarly, several cost-effectiveness studies between SK and TNK, which based their analysis on long-term (≥one year) mortality and outcome, concluded that TNK is better than SK [11,78-81]. On the contrary, GISSI-2 and ISIS-3 trials reported similar one-year mortality between SK and fibrin-specific agents; however, the fibrin-specific agents outperformed SK in younger patients, anterior MI, and previous use of SK [82,83]. Regarding 30-35-day mortality, the available evidence also reported inconsistent findings; a meta-analysis by Jinatongthai et al. concluded that SK is associated with higher 30-35 days mortality [16], while two systematic reviews by Dundar et al. and Ascef et al. reported similar risk of 30-35 days mortality between the two thrombolytics [23,76]. A meta-analysis by Tourani et al. also reported similar mortality; however, the duration was not reported [77].

A clear majority of the studies reported a higher risk of hypotension, allergy, and anaphylaxis with SK. It corresponds with the conclusions of most literature. A meta-analysis by Dundar et al. reported a higher risk of allergic reaction with SK [23], which also corresponds with the findings of a randomized, double-blind, nine-country study: the International Joint Efficacy Comparison of Thrombolytics trial and GUSTO trial [84,85]. Two studies in high-income countries (Hong Kong) also reported a higher risk of hypotension with SK [74,75]. Studies have shown that the severe allergic reaction associated with SK compared to other fibrinolytics is due to its antigenic characteristics [56,85]. According to this study, the evidence is conflicting regarding arrhythmia and re-infarction, though TNK recorded a higher rate of reinfarction while SK recorded a greater risk of arrhythmia. A systematic review by Ascef et al. reported a comparable rate of reinfarction between SK and TNK [76].

Adverse reactions were not synthesized because only two studies recorded them, and the type of adverse reaction was not specified. The two studies reported a higher rate of adverse effects in the SK arm, which corresponds with the report of McCormick et al. [74]; however, it disagrees with the findings of a systematic review by Ascef et al., which reported a similar rate of adverse events among all fibrinolytics [76]. The investigators concluded that TNK is better than SK in symptom relief and complete ST-segment resolution. However, this finding disagrees with the result of a meta-analysis by Dundar et al., which reported similar ST-segment resolution between the two agents [23]. In terms of no ST-segment resolution, there was no clear majority; the findings were conflicting. According to this review, the rate of successful fibrinolysis was comparable between SK and TNK. This finding, however, disagrees with the conclusions of studies conducted in some high-income countries. McCormick et al. concluded in their research carried out in Hong Kong that better reperfusion was achieved with TNK [74]. Similarly, the study by Serpytis et al. carried out in Lithuania reported better fibrinolysis with TNK [66]. Although failed fibrinolysis was not analyzed due to the limited number of studies that reported it (only two), surprisingly, it did not correlate with successful fibrinolysis and ST-segment resolution. The two studies suggested that TNK has more failed fibrinolysis, despite being better with symptom relief and ST-segment resolution.

Points to consider when making a choice between TNK and SK/important gaps in the literature/inferences

In addition to TNK's superiority over SK regarding bleeding, long-term mortality, hypotension, allergy, anaphylaxis, symptom relief, and ST-segment resolution, another essential benefit of TNK is that it is easy to administer [86]. It is administered as a single bolus within 5-10 seconds, while SK is administered intravenously over 30-60 minutes [86]. TNK also has a longer half-life than SK. These are relevant benefits to be considered when choosing fibrinolytic in resource-poor nations, as most patients in these low- and middle-income countries travel long distances to get PCI done [11].

On the other hand, patients who suffered MI in the last year and received an unknown fibrinolytic agent should not be treated with SK because of decreased effectiveness associated with reusing SK and severe allergic reactions/anaphylaxis [82,83]. Hemodynamically unstable patients should not receive SK due to the aforementioned side effects.

Rapid infusion of thrombolytics is the widely preferred method of treating patients with extensive MI [44,82]. Extensive MI is an independent prognosticator of mortality and sudden cardiac death [66]. The prolonged infusion rate of SK makes it unsuitable in cases of extensive MI, which requires rapid infusion.

Another critically important point to consider is that most patients in the emerging world cannot afford PCI [11]. Therefore, most sign up for discharge against medical advice once their chest pain symptom is relieved by the fibrinolytics. TNK has better survival benefits, according to this review; however, only three studies reported one-year mortality; hence, more studies comparing the long-term survival benefit of these agents are essential.

Most studies on cost-effectiveness base their analysis on long-term mortality, disabilities following adverse reactions, quality-adjusted life years, etc., and most suggest that TNK is more cost-effective than SK [11,78-81]. More studies on the cost-effectiveness of fibrinolytic agents are necessary for developing countries as this would be handy while formulating treatment guidelines and insurance/reimbursement policies. In some developing countries (e.g., Thailand), the insurance policy does not cover the use of TNK (due to cost) in managing MI except in cases of allergy to SK [11]. Patients are only reimbursed for SK as it is made the first-line agent in treating MI; thus, a more significant majority of patients are treated with SK in these regions (98% in Thailand) [11] Thus, RCTs and further research on the cost-effectiveness of fibrinolytics are needed in low- and middle-income countries to assist physicians when making treatment decisions and the government when making insurance policies.

Strengths of this study

This study was reported according to the 2020 PRISMA template and was synthesized following Cochrane's guidelines for SWIM. When published, it will fill an essential gap in the literature, the first systematic review to compare the efficacy of TNK VS SK in the emerging world. It is the most comprehensive systematic review of TNK vs SK; seven databases and the reference list of eligible articles, including systematic reviews, were screened. Additionally, six efficacy endpoints and safety parameters were analyzed. It reported long-term mortality, which other systematic reviews on TNK vs SK did not analyze. It also revealed a critical gap in the literature: the lack of multicenter RCTs with adequate sample sizes comparing TNK and SK in this era of dual antiplatelet therapy and anticoagulants.

Limitations of this study

The majority of the studies are observational studies; the diverse baseline patient and clinical characteristics may have affected the result [87].

Confounding factors pose a significant challenge in observational studies [87]. Besides the type of fibrinolytic agent chosen for the management of MI, other confounding factors like door-needle time, comorbidities, the extent of infarction, the territory involved in the infarction, the Killip class, misdiagnosis of STEMI, female gender, delayed presentation, inferior MI, etc. contribute to the outcome of fibrinolytic therapy [88]. These and some unknown factors may have affected the outcome of fibrinolysis. Multiple statistical methods are used to control confounding factors and adjust imbalances between the two arms of an investigation; however, residual confounding still exists as none of the statistical methods can completely account for unknown confounders [87].

Although successful randomization removes known and unknown confounding factors [87], this review has only two RCTs with small sample sizes, thus limiting the study's power.

Additionally, in observational studies, the selection of fibrinolytics is at the treating physician's discretion, thus introducing the risk of selection bias, reporting bias, and publication bias [87].

The small sample size of some included studies limits the study power and questions the generalizability of the outcome. The relatively fewer patients in the TNK arm in most of the study precludes robust comparison, making bias plausible.

The absence of patient-level data in retrospective observational precludes weighted comparison leading to the possibility of bias [87].

Only three studies reported one-year mortality. Research on the long-term outcome of fibrinolytics is fundamental as most patients in resource-poor nations cannot afford PCI; thus, they go home after receiving the fibrinolytics without undergoing PCI.

Research in context

Evidence Before This Study

PUBMED/WEB ff Science was searched for published systematic reviews and meta-analyses applying the following search terms tenecteplase/TNK, streptokinase/SK, and myocardial infarction/STEMI). Four systematic reviews were retrieved [16,23,76,77].

A meta-analysis that was published in 2018 compared the efficacy and safety of TNK vs SK; however, only four observational studies published between 2012 and 2014 were analyzed. It was also published in non-English languages [77].

Another published meta-analysis of RCTs published in 2017 involved data from the Western world and compared all fibrinolytics. There was no direct comparison between TNK and SK. Conclusions were made from extrapolations. The meta-analysis utilized data from trials published between 1985 and 2005, before the era of dual antiplatelet therapy and anticoagulants [16]. Treatment methods have since changed according to current guidelines.

In 2016, a systematic review of three systematic reviews, two health technology assessments, and two therapeutic guidelines on SK, alteplase, and TNK was published; however, it was analyzed in the context of the developed countries, and it didn't establish a hierarchy of superiority among the three agents [76].

The meta-analysis of 14 randomized controlled studies by Dundar et al. published in 2003 did not establish which fibrinolytic is superior in terms of safety and efficacy. It also included data from developed countries. Included studies were published between 1985 and 1999 [23].

None of the systematic reviews commented on long-term mortality (≥1 year), which is vital as the majority of patients in developing countries cannot afford PCI after receiving fibrinolytics.

Added Value of This Study

This study is the first systematic review comparing the efficacy and safety of TNK vs SK in developing countries. It added new data from 13 studies carried out in low- and middle-income countries to the data of the 2018 meta-analysis.

Clinical Implication of This Study

The study established that TNK has better ST-segment resolution and symptom relief compared to SK. It also established that SK had a higher risk of both minor and major bleeding, long-term mortality, hypotension, and allergy/anaphylaxis. The study is a pointer that RCTs with a large sample size are necessary to confirm which lytic has better mortality benefit and less incidence of stroke. SK should be avoided in patients with hemodynamic instability, previous hypersensitivity reactions to SK, and those who received it in the last year [82,83]. Due to the high acquisition cost of TNK and antigenicity of SK, studies on cost-effectiveness are needed in low- and middle-income countries while formulating guidelines for treatment.

It is important to note that most of the articles used in this study are observational studies with small sample sizes and fewer patients in the TNK arm. The RCTs also have a small sample size. Hence, the problem of confounding factors, low study power, and lack of robust comparison between the two fibrinolytic exist. Thus, this study should be interpreted in the light of these limitations.

## Conclusions

According to this narrative synthesis, TNK is more efficacious than SK regarding symptom relief and complete ST-segment resolution. Both agents are similar in terms of successful fibrinolysis. There is conflicting evidence regarding no ST-segment resolution and reinfarction. Concerning safety parameters, SK has a higher risk of bleeding, one-year mortality, hypotension, allergy, and anaphylaxis. Controversy exists regarding 30-day mortality risk, arrhythmias, and hemorrhagic stroke. Only two studies reported failed fibrinolysis and adverse reaction; hence, they were excluded from the narrative synthesis.

RCTs with large sample sizes are needed to establish the effectiveness and safety of TNK vs SK. Studies on cost-effectiveness in developing countries are also essential to confirm which agent is less expensive in the long term. This will enable the government to provide insurance for the most effective thrombolytic agent, irrespective of the acquisition cost.
